# Does stereotype threat influence age-related differences on directed forgetting tasks?

**DOI:** 10.3389/fpsyg.2023.1296662

**Published:** 2024-01-19

**Authors:** Jessie Chih-Yuan Chien, Teal S. Eich

**Affiliations:** The Leonard Davis School of Gerontology, University of Southern California, Los Angeles, CA, United States

**Keywords:** directed forgetting, stereotype threat, memory, aging, inhibtion

## Abstract

**Objectives:**

The Directed Forgetting paradigm has proven to be a powerful tool to explore motivated forgetting in the lab. Past work has shown that older adults are less able to intentionally suppress information from memory relative to younger adults, which is often attributed to deficits in inhibitory abilities. Instructions in traditional Directed Forgetting tasks contain terms that may elicit stereotype threat in older adults, which may negatively impact memory. Here, we tested whether the instructions in a Directed Forgetting task affected older adults’ ability to appropriately control the contents of memory.

**Methods:**

In two experiments that differed in the number of words presented (30 vs. 48 items), younger and older adults were randomized into one of four crossed Conditions of a Directed Forgetting task. At encoding, participants were either instructed to remember/ forget items, or to think about/not think about items. At test, they were either asked whether the memory probe was old or new, or whether they had seen it before (yes/no). Each experiment contained data from 100 younger (18– 40 years) and 98 older (60+ years) adults, with ~25 participants per Condition. All participants were recruited from Prolific and tested online.

**Results:**

In neither Experiment 1 nor Experiment 2 did we find evidence of a stereotype threat effect, or age-related effects of directed forgetting. We did find that performance for to-be-forgotten items was worse in conditions with encoding instructions that contained words that might trigger stereotype threat relative to conditions that did not contain such words: when explicitly told to forget items, both older and younger adults forgot more items than did participants who were cued to not think about the words and put them out of mind. However, we found no such difference across the two different remember instructions: regardless of whether participants were told to remember or to think about items, recognition memory for to be retained items was high. The pattern of results across the two experiments was similar, except, not surprisingly, participants performed worse in Experiment 2 than Experiment 1. Interestingly, we found that higher accuracy for to be remembered items was associated with a more positive outlook of one’s own memory relative to others.

**Discussion:**

These results suggest that directed forgetting may not always be impaired in older adults.

## 1 Introduction

In the intricate web of human cognition, memory stands as a pillar of our mental landscape, enabling us to retain, recall, and make sense of our experiences. While memory is critical in shaping our identities, being able to selectively forget information when we want to is an equally essential but often underappreciated cognitive ability (Bjork, 2014). The adaptive process of forgetting allows us to filter out irrelevant details, prioritize pertinent information, and streamline cognitive resources for optimal functioning. In the lab, the ability to intentionally discard or prioritize memories is often studied using the Directed Forgetting paradigm (Basden and Basden, 1996). In the typical item-method Directed Forgetting task, participants are presented with items one at a time, and are explicitly told to either remember or forget each for a later memory test. The ability to cognitively control the contents of memory – remembering remember items, forgetting forget items -- is measured by presenting participants with both the to be remembered (TBR) items and the to be forgotten (TBF) items, as well as new items, and having them decide whether each probe is old (that is, it was presented during study phase regardless of whether it was a TBR or TBF item) or new. Younger adults typically show poorer memory for the TBF items relative to the TBR items, suggesting that they can, indeed, forget items when explicitly told to do so. A large meta-analysis found age-related differences in this ability (Titz and Verhaeghen, 2010): while older adults show a directed forgetting effect, they drop fewer TBF items from memory than do younger adults, resulting in a smaller difference score between TBR and TBF items. The failure to appropriately drop these items thought to reflect an age-related decline in inhibitory control (Zacks et al., 1996; Anderson and Green, 2001; Hasher and Zacks, 2006; Aguirre et al., 2017; Eich et al., 2018). Our group recently found that this age-related difference was mediated by cortical thickness in the inferior frontal gyrus (Eich et al., 2021), a brain area critical for inhibitory control (Aron et al., 2004, 2014; Eich et al., 2017), suggesting that morphological changes that occur even in healthy aging may have subtle, but consequential effects on cognition.

While changes to inhibitory control may play a role in age-related differences in memorial processes, other factors may also contribute to the differences found between older and younger adults in the traditional Directed Forgetting task. A large literature indicates that one factor that can influence memory is stereotype threat (Steele, 1997). Stereotype threat is a psychological phenomenon that occurs when individuals are exposed to, or made aware of, stereotypes that may apply to them. This awareness or concern of the stereotype causes fear or anticipation of confirming it, which negatively impacts cognitive performance on tasks congruent with the negative stereotype. In the case of aging, the most prevalent stereotype is inevitable age-related memory loss (Hummert et al., 1994; Wurm et al., 2007). Numerous studies have now demonstrated that when older adults are exposed to negative age-related stereotypes, they exhibit poorer memory recall and cognitive functioning (Stein et al., 2002; Hess et al., 2003; Chasteen et al., 2005; Levy, 2009; Eich et al., 2014; Barber, 2017).

In the current study, we were interested in whether the instruction terms “remember,” “forget” and “old” in the traditional Directed Forgetting task may provoke a stereotype threat for older participants, and negatively impact their ability to intentionally remember and forget information. To test this, we randomized younger and older participants to receive encoding and recognition instructions that did or did not contain these terms. One set of encoding and recognition instructions included the terms from the traditional Directed Forgetting task: at encoding participants were told to “Forget” or “Remember” items, and at test they were asked whether the probes were “old” or “new.” The other set were taken from a different task often used to study inhibitory control in memory, the Think/No Think task. The Think/No Think, unlike the Directed Forgetting task, does not contain these potentially age-related memory loss stereotyped terms, and instead instructs participants to “Think” or “Not think” about items for a later memory test, and then indicate whether each probe had been seen before (by making a “Yes” or “No” decision). We crossed these instructions across four conditions, yielding a balanced design that would allow us to investigate differences in the impact of threat related information from encoding versus retrieval.

We predicted that stereotype threat effects, if present, would manifest for older adults in the experimental conditions containing terms related to age-related stereotypes (e.g., “Remember/Forget”; “Old”), and affect memory for TBR items. That is, older adults would show decreased TBR accuracy in stereotype threat conditions. We have previously shown that the ability to inhibit items from memory when instructed to do so is compromised in older individuals, even when episodic memory for to be remembered items is spared (Corlier and Eich, 2022). We speculate that the executive control function of inhibiting information is either more difficult or declines earlier in aging. Based on this, coupled with previous findings from Schmader et al. (2008) who showed that stereotype threat increases cognitive demands, we also predicted that, if present, stereotype threat effects would lead to a greater impairment of TBF items relative to the potential decrement in TBR items resulting from the threat. Finally, we predicted that we would replicate previous findings of a reduced directed forgetting effect (TBR-TBF) for the older as compared to the younger adults.

## 2 Methods

### 2.1 Behavioral tasks

Each of four tasks was modeled after the original item-method Directed Forgetting task (Bjork, 1972), and was administered to participants online using the Gorilla Experiment Builder (www.gorilla.sc; Anwyl-Irvine et al., 2020). Participants were first presented words, one at a time in the center of the screen for 3,000 ms, followed by a 500 ms delay, followed by a memory cue for 1,500 ms. In Conditions 1 and 2, a memory cue, consistent with that presented in a typical Directed Forgetting paradigm, was shown: Participants were told that after each word, they’d see a cue telling them to either remember the word they just saw, or to forget it. When they saw “RRRR” after a word, they should REMEMBER that word. When they saw “FFFF” after a word, they should FORGET that word. They were then told that after all the words had been presented, they would get a memory test for only the words they were told to remember. The instructions for Conditions 3 and 4 were modeled after traditional Think/No Think paradigms (Anderson and Green, 2001). In the Think/No Thin Conditions, participants were told that if they saw “THINK” after a word, they should think about what the word means. When they saw “NO THINK” after a word, they should put the word out of their mind, like it never existed. These elaborate forget instructions were adapted from (Demaine, 2008). Participants were then told that once they’d seen all the words, they’d then be tested on only the words that they were supposed to think about. In all Conditions, half of the words were those that should have been remembered or thought about (for simplicity, we will refer to these as TBR items henceforth) and the other half were those that should have been forgotten or not thought about (for simplicity, we will refer to these as TBF items henceforth). Each memory cue was followed by a 1,000 ms inter trial interval.

Immediately following the presentation of all of the words, participants were presented with these same words again, plus an equal number of new words, in a randomized order. In Conditions 1 and 3, participants were told that for each word, they needed to indicate whether the word was an OLD word (that was shown to them before), or a NEW word (that wasn’t shown to them before). In Conditions 2 and 4, participants were told that for each word they should indicate whether they saw the word before. They should press YES if they had seen it before, and press NO if they had not seen it before. The recognition phase was self-paced, with a maximum of 10 s per item.

Thus, Conditions differed in both the encoding instructions and recognition instructions, yielding a balanced design (Condition 1: Remember or Forget/Old or New; Condition 2: Remember or Forget/Seen or Not Seen; Condition 3: Think or No Think/Old or New; Condition 4: Think or No Think /Seen or Not Seen). The stimuli were drawn from a pool of 100 highly unrelated and unambiguous concrete nouns, ranging in length from three to eight letters. Younger and older adults were randomized into Condition by the software.

In Experiment 1, 30 words were presented at encoding, with half being TBR and the other half TBF items. Participants were then tested on a total of 60 words, 30 of which were the previously presented TBR and TBF words, and 30 were new. We found that performance was near ceiling, and no age-related differences were found even in memory for TBR items. To ensure that these results were not due to the list length being too short, and thus the task too easy, we conducted a second Experiment that was identical to the first, except this time we increased the set size to 48 words, with 24 TBR and 24 TBF items. At test, participants made recognition decisions about 96 items, 48 of which were previously presented, and 48 of which were new.

Following completion of the task, participants were asked basic demographics questions, including how they view their own health and how they view their memory, each rated on a 5-point Likert scale (1. Excellent, 2. Good, 3. OK, 4. Poor, and 5. Terrible). They also rated how they viewed their health and, separately, memory, as compared to someone their age on a 5-point scale (1. Much better, 2. A little better, 3. The same as, 4. A little worse, and 5. Much worse). In Experiment 2, participants additionally completed a standard measure of working memory that has been previously used in large scale online studies that included participants across the lifespan (Logie and Maylor, 2009). In this task, participants saw a rectangular matrix made up of white and blue squares for 2 s. The matrix was then immediately replaced with a blank matrix, and participants had to click, in any order, where the blue squares had been. The matrix increased in size from 3 × 3 (5 blue squares), to 3 × 4 (6 blue), to 4 × 4 (8 blue), to 4 × 5 (10 blue), to a maximum of 5 × 5 (12 blue), with two patterns shown at each level. The task stopped when participants failed to recall all of the squares correctly on two trials at a given matrix size. Performance was scored as the number of patterns correctly recalled (range 0–10). Additionally, to more accurately measure perceptions about age-related changes to cognitive abilities, participants also completed the 12-item Expectations Regarding Aging (ERA) scale (Sarkisian et al., 2005). This scale, which was developed from the full 38-item scale (Sarkisian et al., 2002) has been shown to reliably measure expectations regarding physical health, mental health, and –of particular importance for the present study given its focus on potential stereotype threat mechanisms of directed forgetting effects in aging-- cognitive function.

### 2.2 Participants

Experiments 1 and 2 each contained one hundred younger adults between the ages of 18 and 40 and ninety-eight older adults aged 60+. All participants were recruited from Prolific and completed the study online. Participants in Experiment 2 could not have also been in Experiment 1. The study was approved by the University of Southern California Institutional Review Board (UP-20-01051). All participants gave consent before participating and were paid at a rate of $10/h. Demographic information for both Experiments is presented in Table 1.

**Table 1:** Demographics.

	Experiment 1		Experiment 2	
	Younger	Older	Younger	Older
*N*	100	98	100	98
Age	24.04 (3.673)	69.878 (5.098)***	25.23 (3.429)	68.745 (3.602)***
Years education	14.929 (2.081)	15.673 (2.548)*	15 (2.184)	14.724 (2.372)
*N* female/male	53/46	51/47	46/53	66/32***
Subjective age	24.179 (7.088)	59.516 (12.398)	26.467 (7.646)	57.378 (11.091)
Health	2.12 (0.782)	2.459 (1.017)*	2.37 (0.787)	2.51 (0.803)
Health relative to others	3.04 (0.963)	2.551 (1.15)**	3.11 (0.875)	2.602 (0.982)***
Memory	2.64 (0.847)	2.561 (0.826)	2.48 (0.847)	2.582 (0.745)
Memory relative to others	0.28 (0.451)	0.378 (0.487)**	2.95 (0.968)	2.653 (0.814)~
Visual pattern span			5.667 (2.254)	3.551 (1.922)***
ERA physical health			33.67 (17.453)	34.354 (19.781)
ERA mental health			62.037 (20.558)	61.054 (21.364)
ERA cognitive function			37.121 (18.065)	39.796 (19.862)
ERA Total			44.276 (14.804)	45.068 (16.101)

Standard deviation shown in parentheses. * *p* < 0.05, ** *p* < 0.01, *** *p* < 0.001, ~ *p* = 0.051.

## 3 Results

### 3.1 Experiment 1

There were equal numbers of males and females in the sample (Student’s *t* < 1), but older adults had more education than did younger adults, *t*(195) = 2.246, *p* = 0.026. Older adults also subjectively rated their health as being worse than did younger adults, Mann-Whitney U = 3982.500, *p* = 0.016, and rated their health as being worse than compared to someone their own age, U = 6103.500, *p* = 0.002. While there were no age-related differences between subjective ratings of memory, *p* > 0.5, older adults, compared to younger adults, rated their memory as being better as compared to someone their own age, U = 6030.500, *p* = 0.003.

Descriptive statistics for performance are shown in Table 2. A repeated measure ANOVA on the proportion correct for the three different recognition Probe Types (New; TBR; TBF) as within subjects factors and Age Group (younger; older) and Condition (1–4) as between subjects factor revealed a significant main effect of Probe Type, *F*(2, 380) = 234.015, *p* < 0.001, *η*^2^ = 0.398. *Post hoc* Bonferroni corrected *t*-tests revealed that accuracy was higher for New items than both TBR items, *t* = 3.486, *p* < 0.001, and TBF items, *t* = 20.234, *p* = 0.011, and TBR item accuracy was higher than TBF item accuracy, *t* = 16.748, *p* < 0.001. There was also a main effect of Condition, *F*(3, 190) = 3.810, *p* = 0.011, *η*^2^ = 0.013. Bonferroni correct *t*-tests revealed that participants on average had lower accuracy rates in Condition 2 relative to both Condition 3, *t* = −2.872, *p* = 0.027, and Condition 4, *t* = −2.857, *p* = 0.027.

**Table 2:** Proportion correct by Probe Type.

		Condition	*N*	Mean	St. dev.	Min	Max
New	Exp 1	1	48	0.87	0.126	0.479	1
		2	51	0.895	0.124	0.542	1
		3	49	0.858	0.147	0.333	1
		4	50	0.84	0.196	0.25	1
	Exp 2	1	53	0.945	0.078	0.7	1
		2	47	0.964	0.068	0.667	1
		3	48	0.944	0.076	0.667	1
		4	50	0.94	0.095	0.633	1
TBR	Exp 1	1	48	0.815	0.146	0.333	1
		2	51	0.788	0.163	0.333	1
		3	49	0.836	0.137	0.333	1
		4	50	0.795	0.164	0.292	1
	Exp 2	1	53	0.913	0.099	0.6	1
		2	47	0.87	0.127	0.533	1
		3	48	0.911	0.1	0.667	1
		4	50	0.895	0.104	0.6	1
TBF	Exp 1	1	48	0.628	0.207	0.125	0.917
		2	51	0.56	0.267	0	1
		3	49	0.694	0.187	0.208	0.958
		4	50	0.559	0.234	0.042	0.875
	Exp 2	1	53	0.616	0.247	0	1
		2	47	0.556	0.244	0.133	1
		3	48	0.713	0.188	0.2	1
		4	50	0.729	0.232	0.067	1
Directed forgetting (TBR-TBF)	Exp 1	1	48	0.188	0.189	−0.208	0.667
		2	51	0.228	0.231	−0.125	0.875
		3	49	0.142	0.155	−0.167	0.542
		4	50	0.236	0.236	−0.208	0.958
	Exp 2	1	53	0.297	0.227	0	0.933
		2	47	0.313	0.23	−0.2	0.867
		3	48	0.199	0.177	−0.133	0.733
		4	50	0.165	0.194	−0.133	0.867

Condition (Encoding instruction; Recognition instruction): 1 (Remember/Forget; Old/New), 2 (Remember/Forget; Yes/No), 3 (Think/No Think; Old/New), 4 (Think/No Think; Yes/No).

**Figure 1:**
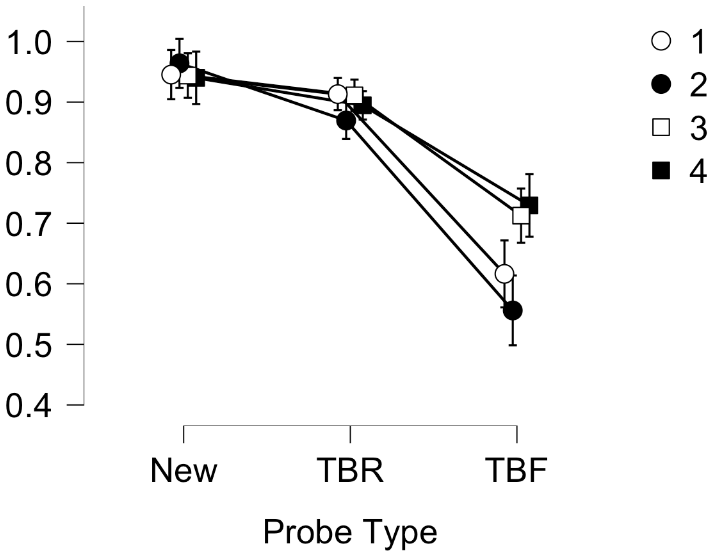
Experiment 1. Proportion correct (y-axis) by Probe Type (x-axis) collapsed across age group for each condition (encoding instructions, recognition instructions) where 1=Remember/Forget, Old/New (white circles); 2=Remember/Forget, Yes/No (black circles); 3=Think/No Think, Old/New (white squares); 4=Think/No think; Yes/ No (black squares). Error bars are standard error of the mean.

We also found a two way interaction between Probe Type and Condition, *F*(1, 190) = 5.585, *p* < 0.001, *η*^2^ = 0.028, which is illustrated in Figure 1. Bonferroni corrected *t*-tests for this interaction are shown in Supplementary Table 1, and revealed that both New and TBR items had higher accuracy rates than did TBF items across all four Conditions. TBF items also had higher accuracy in Condition 1 than in Condition 4. TBF items in Condition 2 were better than they were in Conditions 3 and 4. The main effect of age was not significant, *F*(1,190) = 1.479, *p* = 0.226, and neither were any of the other interactions (Fs < 2).

We also computed an ANOVA on the directed forgetting effect (TBR-TBF), which is thought to be an index of inhibitory control, such that a larger difference between these two types of probes reflects the ability to appropriately drop from memory irrelevant (TBF) items (Hasher et al., 2007). First, we explored only the “traditional” Directed Forgetting task (Condition 1) in an attempt to replicate previous findings showing higher directed forgetting effect scores for older relative to younger adults, which is suggestive of an impairment of inhibitory abilities. Surprisingly, directed forgetting effect values were almost identical for younger and older adults (0.284 vs. 0.310), and were not significantly different from each other, *F*(1, 51) = 0.175, *p* = 0.677. We also investigated the directed forgetting effect including Condition as a between subjects factor. We found a main effect of Condition, *F*(3, 190) = 5.929, *p* < 0.001, *η*^2^ = 0.013. Bonferroni corrected *t*-tests revealed that the directed forgetting effect was larger in Condition 1 relative to Condition 3, *t* = 3.205, *p* = 0.010, and Condition 2 relative to Conditions 4, *t* = 3.461, *p* = 0.004. Neither the main effect of age group, nor the interactions between age group and condition were significant (Fs < 2).

### 3.2 Experiment 2

There were overall more females than males in the second experiment, t(196) = 16.070, *p* < 0.001. Within the younger adults, there were more males than females, t(99) = 9.370, *p* < 0.001, whereas within the older adult group, it was the opposite, t(96) = 13.964, *p* < 0.001. The number of years of education did not differ between younger and older adults, t(193) < 1, and older and younger adults reported equivalent levels of health, U = 4400.500, *p* = 0.180 and memory, U = 4525.500, *p* = 0.318. However, older adults were more likely than younger adults to rate their health as being worse as compared to someone their own age, U = 6317.500, *p* < 0.001, and rated their memory as being marginally better as compared to someone their own age, U = 5636.500, *p* = 0.051. We also found a significant difference in visual pattern span performance between younger and older adults, *t*(7.085), *p* < 0.001, such that younger adults had longer spans. However, there were no significant age-related differences in any of the 4 measures derived from the ERA questionnaire (physical health, mental health, cognitive function or total all ts < 1).

Descriptive statistics for performance are shown in Table 2. A repeated measure ANOVA on Probe Type proportion correct (New; TBR; TBF) as within subjects factors and Age Group (younger; older) and Condition (1–4) as between subjects factor revealed a significant main effect of Probe Type, *F*(2, 380) = 122.199, *p* < 0.001, *η*^2^ = 0.26. *Post hoc* Bonferroni corrected *t*-tests revealed that accuracy was higher for New items than both TBR items, *t* = 3.450, *p* = 0.002, and TBF items, *t* = 14.930, *p* < 0.001, and TBR item accuracy was higher than TBF item accuracy, *t* = 11.480, *p* < 0.001. There was also a main effect of Condition, *F*(3, 190) = 3.702, *p* = 0.013, *η*^2^ = 0.016. Bonferroni correct *t*-tests revealed that participants on average had higher accuracy rates in Condition 3 relative to Condition 4, *t* = 2.872, *p* = 0.027.

**Figure 2:**
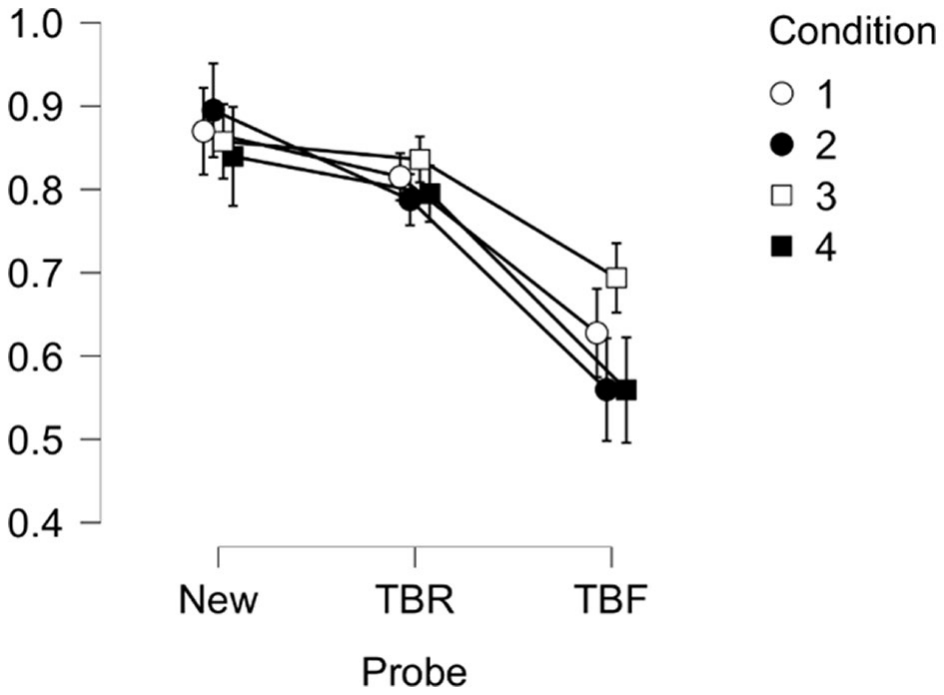
Experiment 2. Proportion correct (y-axis) by Probe Type (x-axis) collapsed across age group for each condition (encoding instructions, recognition instructions) where 1=Remember/Forget, Old/New (white circles); 2=Remember/Forget, Yes/No (black circles); 3=Think/No Think, Old/New (white squares); 4=Think/No think; Yes/ No (black squares). Error bars are standard error of the mean.

We also found a two way interaction between Probe Type and Condition, *F*(6, 380) = 2.437, *p* = 0.025, *η*^2^ = 0.016, which is illustrated in Figure 2. Bonferroni corrected *t*-tests for this interaction are shown in Supplementary Table 2, and were the same as those found in Experiment 1, except that in Experiment 2, TBR items in Conditions 2 and 4 were not better than TBF items in Condition 3, but TBF items were better in Condition 2 than 3, which was better than TBF in Condition 4.

While the results of Experiment 2 largely replicate that of Experiment 1, in this version which contained a longer word list, we did find a significant main effect of age group (*F*(1,190) = 17.268, *p* < 0.001, *η*^2^ = 0.025), such that overall, older adults had lower accuracy than did younger adults. However, none of the interactions with Age Group were significant.

We also computed an ANOVA on the directed forgetting effect (TBR-TBF). First, like before, we explored only the “traditional” Directed Forgetting task (Condition 1). Again, like before, we were surprised to see that, even with a much longer word list, directed forgetting values were almost identical for younger and older adults (0.182 vs. 0.195), and were not significantly different, *F*(1, 46) = 0.051, *p* = 0.823. We also investigated the directed forgetting effect including Condition as a between subjects factor. Unlike in Experiment 1, in Experiment 2 we did not find a main effect of Condition, *F*(3, 190) = 1.999, *p* = 0.115. Neither the main effect of Age Group, nor the interactions between Age Group and Condition were significant (Fs < 2).

### 3.3 Comparison of experiments 1 and 2

The results of Experiment 2 largely replicate those of Experiment 1. However, to directly test this, we added Experiment as a between subjects factor into a repeated measures ANOVA. Here, we found main effects of Age (*F*(1,380) = 15.557, *p* < 0.001, *η*^2^ = 0.010) such that younger adults performed better than older adults, Condition (*F*(3,380) = 4.990, *p* = 0.002, *η*^2^ = 0.009) such that performance was better in Condition 3 than 2 (*t* = −3.843, *p* < 0.001, Bonferroni corrected), Probe Type (*F*(2,760) = 335.936, *p* < 0.001, *η*^2^ = 0.314) such that accuracy for New items was higher than for both TBR (*t* = 4.889, *p* < 0.001) and TBF (*t* = 24.489, *p* < 0.001) items and accuracy was higher for TBR items relative to TBF items, *t* = 19.600, *p* < 0.001, and Experiment, F(1,380) = 47.087, *p* < 0.001, *η*^2^ = 0.030, such that accuracy was worse in Experiment 2 than in Experiment 1. The only significant interaction was between Condition and Probe Type, *F*(6,760) = 5.491, *p* < 0.001, *η*^2^ = 0.015. Bonferroni corrected post-hoc contrasts for this interaction are shown in Supplementary Table 3.

We noted that the directed forgetting effect was numerically smaller in Experiment 2 than in Experiment 1. To explore this, we also analyzed the directed forgetting effect (TBR-TBF) across Experiment, Condition and Probe Type. Indeed, we found a main effect of Experiment, *F*(1, 380) = 4.965, *p* = 0.026, *η*^2^ = 0.012 and a significant main effect of Condition, *F*(3,380) = 4.138, *p* < 0.007, *η*^2^ = 0.030, with Bonferroni corrected post-hoc comparisons revealing that the directed forgetting effect was significantly higher in Condition 2 than in Condition 3 (*t* = 2.448, *p* = 0.009). However, these main effects were qualified by a significant interaction between the two, F(3,380) = 3.784, *p* = 0.011, *η*^2^ = 0.027. Bonferroni corrected post-hoc contrasts for this interaction are shown in Table 3.

**Table 3:** *Post hoc* comparisons of the directed forgetting effect – experiment * condition.

		Mean Difference	SE	*t*	p _bonf_
1, 1	2, 1	0.108	0.042	2.589	0.280
	1, 2	−0.015	0.042	−0.361	1.000
	2, 2	0.077	0.041	1.874	1.000
	1, 3	0.098	0.041	2.375	0.505
	2, 3	0.155	0.041	3.781	0.005**
	1, 4	0.132	0.041	3.222	0.039*
	2, 4	0.061	0.041	1.498	1.000
2, 1	1, 2	−0.123	0.043	−2.862	0.124
	2, 2	−0.031	0.043	−0.739	1.000
	1, 3	−0.010	0.043	−0.235	1.000
	2, 3	0.047	0.043	1.107	1.000
	1, 4	0.023	0.042	0.554	1.000
	2, 4	−0.047	0.042	−1.109	1.000
1, 2	2, 2	0.092	0.042	2.172	0.853
	1, 3	0.113	0.043	2.656	0.231
	2, 3	0.170	0.042	4.021	0.002**
	1, 4	0.147	0.042	3.480	0.016*
	2, 4	0.076	0.042	1.808	1.000
2, 2	1, 3	0.021	0.042	0.507	1.000
	2, 3	0.079	0.042	1.879	1.000
	1, 4	0.055	0.042	1.320	1.000
	2, 4	−0.016	0.042	−0.375	1.000
1, 3	2, 3	0.057	0.042	1.358	1.000
	1, 4	0.034	0.042	0.800	1.000
	2, 4	−0.037	0.042	−0.881	1.000
2, 3	1, 4	−0.024	0.042	−0.568	1.000
	2, 4	−0.094	0.042	−2.258	0.686
1, 4	2, 4	−0.071	0.041	−1.700	1.000

Results are averaged over the levels of age group. *p*-value adjusted for comparing a family of 8. * *p* < 0.05, ** *p* < 0.01.

## 4 Discussion

In two experiments, we explored whether the often-reported age-related differences in the ability to deliberately forget information in Directed Forgetting tasks is influenced by the task instructions, which may emphasize aspects related to negatively held views of age-related memory loss (e.g., “forget,” “old”) and thus provoke an age-related stereotype threat effect.

In Experiment 1, accuracy for both new items and to be remembered (TBR) items was near ceiling across all conditions, and did not differ by age group. Experiment 2, which contained a longer word list, somewhat dampened this ceiling effect, and revealed overall age-related differences in performance, in line with previous literature showing age-related deficits in recognition memory (Spencer and Raz, 1995; Fraundorf et al., 2019). However, somewhat surprisingly, in neither experiment did we replicate previous findings of an age-related difference in Condition 1 of the study, which was identical to traditional Directed Forgetting paradigms where participants are instructed to remember or forget information and then make an old/new recognition decision to memory probes (Zacks et al., 1996; Titz and Verhaeghen, 2010).

Across two experiments, we also did not find evidence for the instructions producing a stereotype threat effect. Indeed, participants in both age groups were more likely to be able to suppress information when they were told, explicitly, to forget it (Conditions 1 and 2), as opposed to when they were told, even with somewhat elaborate instructions, to “put the word out of mind like it had never been there” in both Think/No Think Conditions (Conditions 3 and 4). While we predicted that ST effects would be higher in the Conditions that emphasized stereotypes related to age related memory loss, it is possible that the TBF items in the Think/No Think Conditions were processed more deeply than the TBF items that participants were explicitly told to forget, because participants in the Think/No Think Conditions might process *all* items in the task at a deeper level, following the instruction. If this were the case, then the relative difference in size of the directed forgetting effect (TBR – TBF accuracy) may not stem as much from inhibition of the TBF items in the DF groups as it does from deeper encoding of the TBF items in the TNT groups. However, in both experiments, we found that the TBR items were not better remembered with Think/No Think instructions (Conditions 3 and 4) compared to the DF instructions (Conditions 1 and 2). Interestingly, however, in exploratory analyses when we compared performance on TBR items in both experiments as a function of Condition, while not significant, *F*(3, 392) = 2.252, *p* = 0.081, *η*^2^ = 0.017, accuracy was numerically lower in both recognition conditions that did not emphasize memory, (e.g., Conditions 2 and 4, which asked participants to indicate whether or not they’d seen the item before). These results suggest that both older and younger adults, at least in the current study, can adaptively guide memory to both remember items they want to remember, and forget items they want to forget.

Our group has previously tested older and younger participants recruited from the community on the Condition 1 task (Eich et al., 2021). Whereas the younger adults in the current study of participants recruited online had directed forgetting (TBR-TBF) scores that were almost identical to the younger adults in our study (0.295 vs. 0.284), older adult scores across our two studies were quite different: 0.154 in the previous study of community dwelling older adults in which we replicated an age-related impairment in directed forgetting ability, vs. 0.310 in the current study of participants recruited online through Prolific where we did not. In Experiment 2, directed forgetting effects were more similar to those produced by the community dwelling elders, although in our online sample, we still did not find a typical age-related directed forgetting difference.

While we can only speculate, it is possible that the population of older adults in the Prolific pool are, in some way, different from participants who take part in in-lab studies. Perhaps, for example, the anonymity afforded through online testing changes the way that stereotype triggers impact a person, mitigating the stakes or evaluative pressure that participants might feel about their performance in the lab such that the potential impact of exposure to the age-related stereotype is decreased or undermined. This, in turn, may have led to them perform on par, memorially, with younger adults. Indeed, the fact that we found age invariance in the more sensitive measure of subjective feelings about physical, mental, and cognitive health from the ERA scale introduced in Experiment 2 supports this possibility.

While many studies have found age-related directed forgetting effects, particularly with the item-method paradigm used, in the current study, it should also be noted that this effects is not always found, even in lab-based settings. Sego et al. (2006), for example, found age-invariance in directed forgetting across two experiments that used the item-method directed paradigm. Gamboz and Russo (2002), likewise, found age equivalence when older and younger adults were instructed to process TBF and TBR words using a deeper, as opposed to a more shallow, level of processing. Berger et al. (2018) investigated directed forgetting for positive, negative and neutral stimuli in younger and older participants split into a young-old and an old-old group, using the traditional Directed Forgetting paradigm. While they reported reduced memory in the oldest participants, they did not find age-related directed forgetting effects when deep encoding strategies were used, suggesting that age-related differences arise in part as a result of how information is encoded at the outset.

Other factors that have been shown to modulate the effects of stereotype threat on memory in aging include education level, and factors related to how much a participant identifies with the group to which the stereotype applies. Andreoletti and Lachman (2004), for example, investigated the moderating effect of education level on memory-based stereotype threat effects, reporting that “those with more education are more resilient when faced with negative age stereotypes about memory and respond positively to counterstereotype information.” Ogletree and Katz (2021) have noted that older adults recruited online are often more educated than their younger counterparts. This was the case in Experiment 1, but not Experiment 2.

Bouazzaoui et al. (2016) found that older adult’s episodic memory performance was moderated by threat, such that those older adults exposed to an age-related memory loss stereotype performed worse than those who were not exposed to such a threat. However, they also found that that those individuals exposed to a stereotype threat reported more memory complaints and less memory efficacy, and further that the effect of stereotype threat on episodic memory performance was mediated by both memory complaints and memory self-efficacy. In our sample, while we did not have memory complaint and efficacy measures that were identical to that of Bouazzaoui et al. (2016), our measure of the participants memory relative to a same-age individual, and the ERA scale introduced in Experiment 2, may be close proxies, reflecting a metacognitive feelings related to the participants perception of their own memorial abilities.

To explore whether either education level or subjective ratings of memory compared to another correlates with memory in the current study, we computed Point-Biserial correlations between performance (proportion correct) on all three Probe Types (New, TBR and TBF) and education (median split into high (16+ years) and low (under 16 years)), and Subjective Memory relative to other individuals of the same age, binarized as better than (scores of 1–2 on the scale) or the same as or worse than (scores of 3–5 on the scale). We chose to include the middle score (3) because we were most interested in whether positive perceptions of memory would buffer against ST effects. We found no significant correlations between performance and education in either Experiment 1 or 2, all *p*s > 0.08. It is possible that our education range was too narrow and skewed to pick up on potential differences. We also did not find significant correlations for TBF and New performance and subjective memory relative to others (*p*s > 0.1). However, we did find that TBR item accuracy was correlated with subjective memory relative to others in Experiment 1 (but not Experiment 2), *r* = 0.177, *p* = 0.012, such that those participants who thought their memories were better than other people the same age as them showed superior memory. We found a stronger result when we excluded “same as” (e.g., ratings of a 3 on the scale) responses, *r* = 0.259, *p* = 0.008, a finding that fit nicely with those of Bouazzaoui et al. (2016, 2020). We found no relationship between either Probe Type or the Directed Forgetting effect and either visual pattern span or any of the four measures of perceptions of aging from the 12-item ERA questionnaire (Pearson *r*s = −0.117–0.001).

## 5 Conclusion

In conclusion, the current study aimed to investigate the possibility that age-related differences in the ability to intentionally suppress certain memories, but accurately retain others, is influenced by the instructions given to participants, which we speculated might produce an age-related stereotype threat. Contrary to expectations, we did not find such evidence: there were no significant age-related differences in memory accuracy across Probe Type (TBR, TBF, or New), and there were no age-related differences across the four different encoding and recognition Conditions which varied by virtue of the instructions used, with some containing terms that emphasize aspects of the stereotype of age-related memory loss (e.g., “forget,” “remember,” and “old”). Additionally, the Directed Forgetting effect (TBR-TBF) was not significantly different across younger and older participants in either Experiment 1 or 2, which differed only in the number of items presented at encoding (and tested at recognition). This deviation from the expected age-related effects may be attributable to the characteristics of the participants recruited through the online platform we used. Further exploration is warranted to better understand these complex interactions between cognitive processes, aging, and self-perception.

## Data availability statement

The raw data supporting the conclusions of this article will be made available by the authors, without undue reservation.

## Ethics statement

The studies involving humans were approved by University of Southern California IRB UP-20-01051. The studies were conducted in accordance with the local legislation and institutional requirements. The participants provided their written informed consent to participate in this study.

## Author contributions

JC: Writing – review & editing. TE: Writing – review & editing, Conceptualization, Data curation, Formal analysis, Funding acquisition, Investigation, Methodology, Project administration, Resources, Supervision, Validation, Writing – original draft.
