# Supplementary material for: Does stereotype threat influence age-related differences on directed forgetting tasks?
Source: Front Psychol. 2024 Jan 19;14:1296662. doi: 10.3389/fpsyg.2023.1296662 (PMC10836358; doi:10.3389/fpsyg.2023.1296662)
Supplement: Supplementary file 1 [file Data_Sheet_1.docx]

| **Supplementary Table 1. Post Hoc Comparisons Experiment 1 - Condition ✻ Probe Type** | | | | | | | | | | | |
| --- | --- | --- | --- | --- | --- | --- | --- | --- | --- | --- | --- |
|  | |  | | **Mean Difference** | | **SE** | | **t** | | **p _bonf_** | |
| 1, New |  | 2, New |  | -0.019 |  | 0.031 |  | -0.608 |  | 1.000 |  |
|  |  | 3, New |  | 0.003 |  | 0.031 |  | 0.096 |  | 1.000 |  |
|  |  | 4, New |  | 0.005 |  | 0.030 |  | 0.179 |  | 1.000 |  |
|  |  | 1, TBR |  | 0.032 |  | 0.028 |  | 1.158 |  | 1.000 |  |
|  |  | 2, TBR |  | 0.075 |  | 0.031 |  | 2.434 |  | 1.000 |  |
|  |  | 3, TBR |  | 0.034 |  | 0.031 |  | 1.109 |  | 1.000 |  |
|  |  | 4, TBR |  | 0.051 |  | 0.030 |  | 1.679 |  | 1.000 |  |
|  |  | 1, TBF |  | 0.330 |  | 0.028 |  | 11.756 |  | < .001 | *** |
|  |  | 2, TBF |  | 0.387 |  | 0.031 |  | 12.590 |  | < .001 | *** |
|  |  | 3, TBF |  | 0.233 |  | 0.031 |  | 7.621 |  | < .001 | *** |
|  |  | 4, TBF |  | 0.216 |  | 0.030 |  | 7.153 |  | < .001 | *** |
| 2, New |  | 3, New |  | 0.022 |  | 0.031 |  | 0.687 |  | 1.000 |  |
|  |  | 4, New |  | 0.024 |  | 0.031 |  | 0.773 |  | 1.000 |  |
|  |  | 1, TBR |  | 0.051 |  | 0.031 |  | 1.665 |  | 1.000 |  |
|  |  | 2, TBR |  | 0.093 |  | 0.030 |  | 3.134 |  | 0.123 |  |
|  |  | 3, TBR |  | 0.053 |  | 0.031 |  | 1.669 |  | 1.000 |  |
|  |  | 4, TBR |  | 0.069 |  | 0.031 |  | 2.228 |  | 1.000 |  |
|  |  | 1, TBF |  | 0.348 |  | 0.031 |  | 11.333 |  | < .001 | *** |
|  |  | 2, TBF |  | 0.406 |  | 0.030 |  | 13.598 |  | < .001 | *** |
|  |  | 3, TBF |  | 0.251 |  | 0.031 |  | 7.986 |  | < .001 | *** |
|  |  | 4, TBF |  | 0.235 |  | 0.031 |  | 7.534 |  | < .001 | *** |
| 3, New |  | 4, New |  | 0.002 |  | 0.031 |  | 0.080 |  | 1.000 |  |
|  |  | 1, TBR |  | 0.030 |  | 0.031 |  | 0.967 |  | 1.000 |  |
|  |  | 2, TBR |  | 0.072 |  | 0.031 |  | 2.282 |  | 1.000 |  |
|  |  | 3, TBR |  | 0.031 |  | 0.029 |  | 1.049 |  | 1.000 |  |
|  |  | 4, TBR |  | 0.048 |  | 0.031 |  | 1.543 |  | 1.000 |  |
|  |  | 1, TBF |  | 0.327 |  | 0.031 |  | 10.695 |  | < .001 | *** |
|  |  | 2, TBF |  | 0.384 |  | 0.031 |  | 12.196 |  | < .001 | *** |
|  |  | 3, TBF |  | 0.230 |  | 0.029 |  | 7.796 |  | < .001 | *** |
|  |  | 4, TBF |  | 0.213 |  | 0.031 |  | 6.881 |  | < .001 | *** |
| 4, New |  | 1, TBR |  | 0.027 |  | 0.030 |  | 0.897 |  | 1.000 |  |
|  |  | 2, TBR |  | 0.069 |  | 0.031 |  | 2.227 |  | 1.000 |  |
|  |  | 3, TBR |  | 0.028 |  | 0.031 |  | 0.919 |  | 1.000 |  |
|  |  | 4, TBR |  | 0.045 |  | 0.029 |  | 1.571 |  | 1.000 |  |
|  |  | 1, TBF |  | 0.324 |  | 0.030 |  | 10.732 |  | < .001 | *** |
|  |  | 2, TBF |  | 0.382 |  | 0.031 |  | 12.244 |  | < .001 | *** |
|  |  | 3, TBF |  | 0.227 |  | 0.031 |  | 7.341 |  | < .001 | *** |
|  |  | 4, TBF |  | 0.211 |  | 0.029 |  | 7.300 |  | < .001 | *** |
| 1, TBR |  | 2, TBR |  | 0.042 |  | 0.031 |  | 1.377 |  | 1.000 |  |
|  |  | 3, TBR |  | 0.001 |  | 0.031 |  | 0.045 |  | 1.000 |  |
|  |  | 4, TBR |  | 0.018 |  | 0.030 |  | 0.604 |  | 1.000 |  |
|  |  | 1, TBF |  | 0.297 |  | 0.028 |  | 10.597 |  | < .001 | *** |
|  |  | 2, TBF |  | 0.354 |  | 0.031 |  | 11.533 |  | < .001 | *** |
|  |  | 3, TBF |  | 0.200 |  | 0.031 |  | 6.557 |  | < .001 | *** |
|  |  | 4, TBF |  | 0.184 |  | 0.030 |  | 6.077 |  | < .001 | *** |
| 2, TBR |  | 3, TBR |  | -0.041 |  | 0.031 |  | -1.300 |  | 1.000 |  |
|  |  | 4, TBR |  | -0.024 |  | 0.031 |  | -0.772 |  | 1.000 |  |
|  |  | 1, TBF |  | 0.255 |  | 0.031 |  | 8.291 |  | < .001 | *** |
|  |  | 2, TBF |  | 0.312 |  | 0.030 |  | 10.464 |  | < .001 | *** |
|  |  | 3, TBF |  | 0.158 |  | 0.031 |  | 5.017 |  | < .001 | *** |
|  |  | 4, TBF |  | 0.141 |  | 0.031 |  | 4.534 |  | < .001 | *** |
| 3, TBR |  | 4, TBR |  | 0.017 |  | 0.031 |  | 0.545 |  | 1.000 |  |
|  |  | 1, TBF |  | 0.296 |  | 0.031 |  | 9.682 |  | < .001 | *** |
|  |  | 2, TBF |  | 0.353 |  | 0.031 |  | 11.213 |  | < .001 | *** |
|  |  | 3, TBF |  | 0.199 |  | 0.029 |  | 6.747 |  | < .001 | *** |
|  |  | 4, TBF |  | 0.182 |  | 0.031 |  | 5.883 |  | < .001 | *** |
| 4, TBR |  | 1, TBF |  | 0.279 |  | 0.030 |  | 9.231 |  | < .001 | *** |
|  |  | 2, TBF |  | 0.336 |  | 0.031 |  | 10.789 |  | < .001 | *** |
|  |  | 3, TBF |  | 0.182 |  | 0.031 |  | 5.877 |  | < .001 | *** |
|  |  | 4, TBF |  | 0.165 |  | 0.029 |  | 5.729 |  | < .001 | *** |
| 1, TBF |  | 2, TBF |  | 0.057 |  | 0.031 |  | 1.865 |  | 1.000 |  |
|  |  | 3, TBF |  | -0.097 |  | 0.031 |  | -3.170 |  | 0.106 |  |
|  |  | 4, TBF |  | -0.114 |  | 0.030 |  | -3.758 |  | 0.012 | * |
| 2, TBF |  | 3, TBF |  | -0.154 |  | 0.031 |  | -4.896 |  | < .001 | *** |
|  |  | 4, TBF |  | -0.171 |  | 0.031 |  | -5.483 |  | < .001 | *** |
| 3, TBF |  | 4, TBF |  | -0.017 |  | 0.031 |  | -0.539 |  | 1.000 |  |
|  | | | | | | | | | | | |
| Note.  Results are averaged over the levels of Age Group | | | | | | | | | | | |
| Note.  P-value adjusted for comparing a family of 66 | | | | | | | | | | | |
| * p < .05, ** p < .01, *** p < .001 | | | | | | | | | | | |

| **Supplementary Table 2. Post Hoc Comparisons Experiment 2 - Condition ✻ Probe Type** | | | | | | | | | | | |
| --- | --- | --- | --- | --- | --- | --- | --- | --- | --- | --- | --- |
|  | |  | | **Mean Difference** | | **SE** | | **t** | | **p _bonf_** | |
| 1, New |  | 2, New |  | -0.013 |  | 0.036 |  | -0.370 |  | 1.000 |  |
|  |  | 3, New |  | 0.015 |  | 0.037 |  | 0.422 |  | 1.000 |  |
|  |  | 4, New |  | 0.034 |  | 0.036 |  | 0.943 |  | 1.000 |  |
|  |  | 1, TBR |  | 0.055 |  | 0.035 |  | 1.548 |  | 1.000 |  |
|  |  | 2, TBR |  | 0.101 |  | 0.036 |  | 2.781 |  | 0.370 |  |
|  |  | 3, TBR |  | 0.038 |  | 0.037 |  | 1.032 |  | 1.000 |  |
|  |  | 4, TBR |  | 0.079 |  | 0.036 |  | 2.168 |  | 1.000 |  |
|  |  | 1, TBF |  | 0.243 |  | 0.035 |  | 6.910 |  | < .001 | *** |
|  |  | 2, TBF |  | 0.322 |  | 0.036 |  | 8.816 |  | < .001 | *** |
|  |  | 3, TBF |  | 0.179 |  | 0.037 |  | 4.906 |  | < .001 | *** |
|  |  | 4, TBF |  | 0.315 |  | 0.036 |  | 8.649 |  | < .001 | *** |
| 2, New |  | 3, New |  | 0.029 |  | 0.036 |  | 0.806 |  | 1.000 |  |
|  |  | 4, New |  | 0.048 |  | 0.036 |  | 1.339 |  | 1.000 |  |
|  |  | 1, TBR |  | 0.068 |  | 0.036 |  | 1.864 |  | 1.000 |  |
|  |  | 2, TBR |  | 0.115 |  | 0.034 |  | 3.387 |  | 0.052 |  |
|  |  | 3, TBR |  | 0.051 |  | 0.036 |  | 1.428 |  | 1.000 |  |
|  |  | 4, TBR |  | 0.092 |  | 0.036 |  | 2.587 |  | 0.655 |  |
|  |  | 1, TBF |  | 0.257 |  | 0.036 |  | 7.038 |  | < .001 | *** |
|  |  | 2, TBF |  | 0.335 |  | 0.034 |  | 9.874 |  | < .001 | *** |
|  |  | 3, TBF |  | 0.193 |  | 0.036 |  | 5.374 |  | < .001 | *** |
|  |  | 4, TBF |  | 0.328 |  | 0.036 |  | 9.191 |  | < .001 | *** |
| 3, New |  | 4, New |  | 0.019 |  | 0.036 |  | 0.527 |  | 1.000 |  |
|  |  | 1, TBR |  | 0.039 |  | 0.037 |  | 1.068 |  | 1.000 |  |
|  |  | 2, TBR |  | 0.086 |  | 0.036 |  | 2.397 |  | 1.000 |  |
|  |  | 3, TBR |  | 0.022 |  | 0.034 |  | 0.654 |  | 1.000 |  |
|  |  | 4, TBR |  | 0.063 |  | 0.036 |  | 1.773 |  | 1.000 |  |
|  |  | 1, TBF |  | 0.228 |  | 0.037 |  | 6.231 |  | < .001 | *** |
|  |  | 2, TBF |  | 0.306 |  | 0.036 |  | 8.532 |  | < .001 | *** |
|  |  | 3, TBF |  | 0.164 |  | 0.034 |  | 4.809 |  | < .001 | *** |
|  |  | 4, TBF |  | 0.299 |  | 0.036 |  | 8.362 |  | < .001 | *** |
| 4, New |  | 1, TBR |  | 0.020 |  | 0.036 |  | 0.555 |  | 1.000 |  |
|  |  | 2, TBR |  | 0.067 |  | 0.036 |  | 1.881 |  | 1.000 |  |
|  |  | 3, TBR |  | 0.003 |  | 0.036 |  | 0.096 |  | 1.000 |  |
|  |  | 4, TBR |  | 0.045 |  | 0.034 |  | 1.321 |  | 1.000 |  |
|  |  | 1, TBF |  | 0.209 |  | 0.036 |  | 5.744 |  | < .001 | *** |
|  |  | 2, TBF |  | 0.287 |  | 0.036 |  | 8.047 |  | < .001 | *** |
|  |  | 3, TBF |  | 0.145 |  | 0.036 |  | 4.054 |  | 0.004 | ** |
|  |  | 4, TBF |  | 0.280 |  | 0.034 |  | 8.309 |  | < .001 | *** |
| 1, TBR |  | 2, TBR |  | 0.047 |  | 0.036 |  | 1.287 |  | 1.000 |  |
|  |  | 3, TBR |  | -0.017 |  | 0.037 |  | -0.458 |  | 1.000 |  |
|  |  | 4, TBR |  | 0.024 |  | 0.036 |  | 0.670 |  | 1.000 |  |
|  |  | 1, TBF |  | 0.189 |  | 0.035 |  | 5.362 |  | < .001 | *** |
|  |  | 2, TBF |  | 0.267 |  | 0.036 |  | 7.322 |  | < .001 | *** |
|  |  | 3, TBF |  | 0.125 |  | 0.037 |  | 3.415 |  | 0.045 | * |
|  |  | 4, TBF |  | 0.260 |  | 0.036 |  | 7.150 |  | < .001 | *** |
| 2, TBR |  | 3, TBR |  | -0.064 |  | 0.036 |  | -1.775 |  | 1.000 |  |
|  |  | 4, TBR |  | -0.023 |  | 0.036 |  | -0.632 |  | 1.000 |  |
|  |  | 1, TBF |  | 0.142 |  | 0.036 |  | 3.887 |  | 0.007 | ** |
|  |  | 2, TBF |  | 0.220 |  | 0.034 |  | 6.487 |  | < .001 | *** |
|  |  | 3, TBF |  | 0.078 |  | 0.036 |  | 2.171 |  | 1.000 |  |
|  |  | 4, TBF |  | 0.213 |  | 0.036 |  | 5.971 |  | < .001 | *** |
| 3, TBR |  | 4, TBR |  | 0.041 |  | 0.036 |  | 1.150 |  | 1.000 |  |
|  |  | 1, TBF |  | 0.206 |  | 0.037 |  | 5.621 |  | < .001 | *** |
|  |  | 2, TBF |  | 0.284 |  | 0.036 |  | 7.910 |  | < .001 | *** |
|  |  | 3, TBF |  | 0.142 |  | 0.034 |  | 4.155 |  | 0.003 | ** |
|  |  | 4, TBF |  | 0.277 |  | 0.036 |  | 7.738 |  | < .001 | *** |
| 4, TBR |  | 1, TBF |  | 0.164 |  | 0.036 |  | 4.519 |  | < .001 | *** |
|  |  | 2, TBF |  | 0.243 |  | 0.036 |  | 6.799 |  | < .001 | *** |
|  |  | 3, TBF |  | 0.101 |  | 0.036 |  | 2.808 |  | 0.340 |  |
|  |  | 4, TBF |  | 0.236 |  | 0.034 |  | 6.988 |  | < .001 | *** |
| 1, TBF |  | 2, TBF |  | 0.078 |  | 0.036 |  | 2.148 |  | 1.000 |  |
|  |  | 3, TBF |  | -0.064 |  | 0.037 |  | -1.748 |  | 1.000 |  |
|  |  | 4, TBF |  | 0.071 |  | 0.036 |  | 1.962 |  | 1.000 |  |
| 2, TBF |  | 3, TBF |  | -0.142 |  | 0.036 |  | -3.964 |  | 0.005 | ** |
|  |  | 4, TBF |  | -0.007 |  | 0.036 |  | -0.196 |  | 1.000 |  |
| 3, TBF |  | 4, TBF |  | 0.135 |  | 0.036 |  | 3.780 |  | 0.011 | * |
|  | | | | | | | | | | | |
| Note.  Results are averaged over the levels of Age Group | | | | | | | | | | | |
| Note.  P-value adjusted for comparing a family of 66 | | | | | | | | | | | |
| * p < .05, ** p < .01, *** p < .001 | | | | | | | | | | | |

| **Supplementary Table 3. Post Hoc Comparisons Experiments 1 and 2 - Condition ✻ Probe Type** | | | | | | | | | | | |
| --- | --- | --- | --- | --- | --- | --- | --- | --- | --- | --- | --- |
|  | |  | | **Mean Difference** | | **SE** | | **t** | | **p _bonf_** | |
| 1, New |  | 2, New |  | -0.016 |  | 0.024 |  | -0.676 |  | 1.000 |  |
|  |  | 3, New |  | 0.009 |  | 0.024 |  | 0.388 |  | 1.000 |  |
|  |  | 4, New |  | 0.020 |  | 0.024 |  | 0.842 |  | 1.000 |  |
|  |  | 1, TBR |  | 0.044 |  | 0.022 |  | 1.947 |  | 1.000 |  |
|  |  | 2, TBR |  | 0.088 |  | 0.024 |  | 3.702 |  | 0.015 | * |
|  |  | 3, TBR |  | 0.036 |  | 0.024 |  | 1.508 |  | 1.000 |  |
|  |  | 4, TBR |  | 0.065 |  | 0.024 |  | 2.751 |  | 0.398 |  |
|  |  | 1, TBF |  | 0.286 |  | 0.022 |  | 12.800 |  | < .001 | *** |
|  |  | 2, TBF |  | 0.354 |  | 0.024 |  | 14.879 |  | < .001 | *** |
|  |  | 3, TBF |  | 0.206 |  | 0.024 |  | 8.670 |  | < .001 | *** |
|  |  | 4, TBF |  | 0.265 |  | 0.024 |  | 11.255 |  | < .001 | *** |
| 2, New |  | 3, New |  | 0.025 |  | 0.024 |  | 1.059 |  | 1.000 |  |
|  |  | 4, New |  | 0.036 |  | 0.024 |  | 1.516 |  | 1.000 |  |
|  |  | 1, TBR |  | 0.060 |  | 0.024 |  | 2.506 |  | 0.816 |  |
|  |  | 2, TBR |  | 0.104 |  | 0.023 |  | 4.605 |  | < .001 | *** |
|  |  | 3, TBR |  | 0.052 |  | 0.024 |  | 2.173 |  | 1.000 |  |
|  |  | 4, TBR |  | 0.081 |  | 0.024 |  | 3.414 |  | 0.044 | * |
|  |  | 1, TBF |  | 0.303 |  | 0.024 |  | 12.705 |  | < .001 | *** |
|  |  | 2, TBF |  | 0.370 |  | 0.023 |  | 16.362 |  | < .001 | *** |
|  |  | 3, TBF |  | 0.222 |  | 0.024 |  | 9.294 |  | < .001 | *** |
|  |  | 4, TBF |  | 0.282 |  | 0.024 |  | 11.869 |  | < .001 | *** |
| 3, New |  | 4, New |  | 0.011 |  | 0.024 |  | 0.449 |  | 1.000 |  |
|  |  | 1, TBR |  | 0.034 |  | 0.024 |  | 1.445 |  | 1.000 |  |
|  |  | 2, TBR |  | 0.079 |  | 0.024 |  | 3.301 |  | 0.065 |  |
|  |  | 3, TBR |  | 0.027 |  | 0.023 |  | 1.180 |  | 1.000 |  |
|  |  | 4, TBR |  | 0.056 |  | 0.024 |  | 2.351 |  | 1.000 |  |
|  |  | 1, TBF |  | 0.277 |  | 0.024 |  | 11.664 |  | < .001 | *** |
|  |  | 2, TBF |  | 0.345 |  | 0.024 |  | 14.436 |  | < .001 | *** |
|  |  | 3, TBF |  | 0.197 |  | 0.023 |  | 8.728 |  | < .001 | *** |
|  |  | 4, TBF |  | 0.256 |  | 0.024 |  | 10.822 |  | < .001 | *** |
| 4, New |  | 1, TBR |  | 0.024 |  | 0.024 |  | 1.005 |  | 1.000 |  |
|  |  | 2, TBR |  | 0.068 |  | 0.024 |  | 2.879 |  | 0.268 |  |
|  |  | 3, TBR |  | 0.016 |  | 0.024 |  | 0.675 |  | 1.000 |  |
|  |  | 4, TBR |  | 0.045 |  | 0.022 |  | 2.028 |  | 1.000 |  |
|  |  | 1, TBF |  | 0.267 |  | 0.024 |  | 11.306 |  | < .001 | *** |
|  |  | 2, TBF |  | 0.335 |  | 0.024 |  | 14.101 |  | < .001 | *** |
|  |  | 3, TBF |  | 0.186 |  | 0.024 |  | 7.866 |  | < .001 | *** |
|  |  | 4, TBF |  | 0.246 |  | 0.022 |  | 11.061 |  | < .001 | *** |
| 1, TBR |  | 2, TBR |  | 0.045 |  | 0.024 |  | 1.872 |  | 1.000 |  |
|  |  | 3, TBR |  | -0.008 |  | 0.024 |  | -0.325 |  | 1.000 |  |
|  |  | 4, TBR |  | 0.021 |  | 0.024 |  | 0.904 |  | 1.000 |  |
|  |  | 1, TBF |  | 0.243 |  | 0.022 |  | 10.853 |  | < .001 | *** |
|  |  | 2, TBF |  | 0.311 |  | 0.024 |  | 13.049 |  | < .001 | *** |
|  |  | 3, TBF |  | 0.163 |  | 0.024 |  | 6.837 |  | < .001 | *** |
|  |  | 4, TBF |  | 0.222 |  | 0.024 |  | 9.408 |  | < .001 | *** |
| 2, TBR |  | 3, TBR |  | -0.052 |  | 0.024 |  | -2.188 |  | 1.000 |  |
|  |  | 4, TBR |  | -0.023 |  | 0.024 |  | -0.981 |  | 1.000 |  |
|  |  | 1, TBF |  | 0.198 |  | 0.024 |  | 8.327 |  | < .001 | *** |
|  |  | 2, TBF |  | 0.266 |  | 0.023 |  | 11.757 |  | < .001 | *** |
|  |  | 3, TBF |  | 0.118 |  | 0.024 |  | 4.933 |  | < .001 | *** |
|  |  | 4, TBF |  | 0.177 |  | 0.024 |  | 7.473 |  | < .001 | *** |
| 3, TBR |  | 4, TBR |  | 0.029 |  | 0.024 |  | 1.226 |  | 1.000 |  |
|  |  | 1, TBF |  | 0.251 |  | 0.024 |  | 10.544 |  | < .001 | *** |
|  |  | 2, TBF |  | 0.319 |  | 0.024 |  | 13.323 |  | < .001 | *** |
|  |  | 3, TBF |  | 0.170 |  | 0.023 |  | 7.548 |  | < .001 | *** |
|  |  | 4, TBF |  | 0.230 |  | 0.024 |  | 9.697 |  | < .001 | *** |
| 4, TBR |  | 1, TBF |  | 0.222 |  | 0.024 |  | 9.396 |  | < .001 | *** |
|  |  | 2, TBF |  | 0.289 |  | 0.024 |  | 12.203 |  | < .001 | *** |
|  |  | 3, TBF |  | 0.141 |  | 0.024 |  | 5.964 |  | < .001 | *** |
|  |  | 4, TBF |  | 0.201 |  | 0.022 |  | 9.033 |  | < .001 | *** |
| 1, TBF |  | 2, TBF |  | 0.068 |  | 0.024 |  | 2.850 |  | 0.294 |  |
|  |  | 3, TBF |  | -0.080 |  | 0.024 |  | -3.382 |  | 0.049 | * |
|  |  | 4, TBF |  | -0.021 |  | 0.024 |  | -0.892 |  | 1.000 |  |
| 2, TBF |  | 3, TBF |  | -0.148 |  | 0.024 |  | -6.202 |  | < .001 | *** |
|  |  | 4, TBF |  | -0.089 |  | 0.024 |  | -3.749 |  | 0.012 | * |
| 3, TBF |  | 4, TBF |  | 0.059 |  | 0.024 |  | 2.507 |  | 0.814 |  |
|  | | | | | | | | | | | |
| Note.  Results are averaged over the levels of Experiment and Age Group | | | | | | | | | | | |
| Note.  P-value adjusted for comparing a family of 66 | | | | | | | | | | | |
| * p < .05, ** p < .01, *** p < .001 | | | | | | | | | | | |
